# Antimicrobial Resistance and Associated Risk Factors for *Clostridium difficile* in Patients Attending Tertiary Care Settings

**DOI:** 10.1155/2024/6613120

**Published:** 2024-05-16

**Authors:** Murad A. Mubaraki, Mubbashir Hussain, Faaiz Ul Hassan, Shahzad Munir, Fozia Fozia, Ijaz Ahmad, Fatima Bibi, Samia Sultan, Ziaullah Zialluh

**Affiliations:** ^1^Clinical Laboratory Sciences Department, College of Applied Medical Sciences, King Saud University, Riyadh, Saudi Arabia; ^2^Department of Microbiology, Kohat University of Science and Technology, Kohat, Pakistan; ^3^Yunnan Agricultural University, Kunming, China; ^4^Department of Biochemistry, KMU Institute of Dental Sciences, Kohat 26000, Pakistan; ^5^Department of Chemistry, Kohat University of Science and Technology, Kohat, Pakistan; ^6^Department of Zoology, Abdul Wali Khan University, Mardan, Pakistan; ^7^College of Professional Studies, Northeastern University, Boston, MA, USA

## Abstract

To determine the incidence of antimicrobial-resistant emerging pathogens, *Clostridium difficile*, and its associated risk factors in tertiary care setups of Pakistan. This cross-sectional prospective study was conducted from January 2019 to December 2020, to determine the prevalence and antimicrobial resistance patterns of *C. difficile* strains isolated from 450 stool specimens of patients suffering from diarrhea hospitalized in tertiary care hospitals in Peshawar, Pakistan. The stool samples of the patients were processed for culture and detection of toxin A and toxin B by enzyme-linked immunosorbent assay (ELISA) and *tpi* PCR. The drug sensitivity test was performed for antibiotics including ampicillin, cefixime, cefepime, amoxicillin, nalidixic acid, sulpha/TMP (SXT), chloramphenicol, metronidazole, vancomycin, ciprofloxacin, levofloxacin, and imipenem. Of 450 stool specimens, 108 (24%) were positive for *C. difficile* by stool culture, whereas 115 (25.5%) were only positive for *C. difficile* toxins based on ELISA and PCR (128 (28.6%). Of 108, 90.7% (*n* = 98) isolates were resistant to one antibiotic, and 90 (83.4%) were resistant to three or more antimicrobials. The highest resistance rates were found against penicillin (83.3%) followed by amoxicillin (70%), nalidixic acid (61%), and metronidazole (38%), and the lowest resistance was found against vancomycin (6.4%) and imipenem (3.7%). CDI was statistically significantly correlated with increased age, use of antibiotics, abdominal surgeries, use of proton pump inhibitors and H2a, and presence of comorbidities. The high frequency of *C. difficile* in Peshawar, Pakistan, indicates that CDI is an important nosocomial infection in different hospitals. The results will be helpful for clinicians to redesign control and therapeutic strategies in hospitals.

## 1. Introduction

*Clostridium difficile (C. difficile)* is an obligate anaerobic, spore-forming bacillus present as a part of gut flora in the intestinal tract of healthy adults and elder subjects. It has recently been renamed as *Clostridioides difficile* [1]. It is a major cause of hospital-associated enterocolitis resulting in antibiotic-associated diarrhea, pseudomembranous colitis antibiotic-associated colitis, sepsis, and even death. These infections are collectively known as *Clostridium difficile* infections (CDI) [2–4]. *C. difficile* is a spore-forming bacterium that can survive in the hospital setting for a long time and can spread in hospitals through contaminated equipment and hospital personnel [5].

CDI spread within hospitals and among communities has made it more challenging than ever. The increased morbidity and mortality among hospitalized patients diagnosed with CDI results in a significant economic burden on healthcare systems in developing and low-income countries [6–8]. Outbreaks of severe CDI have been caused by the hypervirulent strain of *C. difficile* various PCR ribotypes, i.e., 027, 014, 020, 106, and 002, in Canada [9], while 078, 014, 012, 020, and 002 were found in Europe [10, 11]. The pathogenesis of CDI is mediated by enterotoxin A and cytotoxin B encoded by *TcdA* and *TcdB* genes [12, 13].

Hospital stays with a history of prolonged antibiotic therapy, irrational use of multiple antibiotics, old age, presence of comorbidities, use of a nasogastric tube, type of gastrointestinal procedures, antiulcer medications, and many others are important risk factors in the development of CDI, and there is a need to explore other possible risk factors that may vary with hospital settings and [14] prolonged antibiotic therapies in healthcare facilities [15].

The spread of multidrug-resistant (MDR) strains of *C. difficile* in hospitals has become a major concern. Vancomycin and metronidazole have been used as a treatment of choice to treat CDI for many years [16]; however, several studies have currently reported reduced rates of susceptibility to these antibiotics as well as to many others [17]. This highlights the need for new antibiotics for the treatment of *C. difficile* strains. Thus, regular surveillance of the antibiogram assays and diagnosis of MDR *C. difficile* strains is important.

The prognosis and therapeutic success of *C. difficile* mainly depend on laboratory identification of *C. difficile* strains, and the detection of toxins is highly important for both conducting surveillance studies [18]. Bacterial culture, toxin enzyme-linked immunosorbent assay (ELISA), and molecular techniques like PCR are frequently used for this purpose. Bacteriological culture is recommended because it helps in the detection of toxigenic isolates in the culture as well as makes available the isolates suitable for toxigenic typing and determination of antimicrobial susceptibility patterns. ELISA is useful to detect toxins *TcdA* and/or *TcdB* in stool specimens [19]. Moreover, molecular identification of *C. difficile* by various PCR techniques is being used frequently [20].

The current study was performed to evaluate the prevalence of antimicrobial-resistant *C. difficile* in stool samples of hospitalized diarrheal patients in different tertiary care hospitals of Peshawar, Pakistan, using techniques like bacteriological culture and toxin A/B confirmation using ELISA test and *Tpi* gene PCR. We believe that this study is an initial step towards the introduction of proper interventions to control CDI in Pakistan.

## 2. Materials and Methods

The cross-sectional prospective study was conducted at three tertiary care hospitals, Peshawar, the capital of Khyber Pakhtunkhwa (KP), Pakistan, from January 2019 to December 2020 and used the STROBE cross-sectional reporting guidelines [21].

### 2.1. Ethics and Dissemination

Ethics approval of the study was obtained from the Ethical and Research Committee, Department of Microbiology, Kohat University of Science & Technology, Kohat, Pakistan (Ref. No. 25/Ethical/MICRO/KUST), on December 18, 2018. Written informed consent was sought from all study participants before sample collection was conducted. In the case of minors (less than 18 years of age), consent was taken from parents, relatives, or guardians. Informed consent specified that the data would be made public in the form of publications.

These hospitals included the Lady Reading Hospital (LRH), Hayat Abad Medical Complex (HMC), and Khyber Teaching Hospital (KTH). These hospitals are the main referral hospitals of Peshawar and receive patients from all over the KP. Sampling was done from different wards of these hospitals including medical (Gastroenterology, Gynecology, Pulmonology, Oncology, Urology) and surgical wards (Cardiology, Surgical, Cardiology, Orthopedics, Neurosurgery), intensive care units (ICUs), and burn and plastic surgery unit. Culture of bacteria and PCR was performed at the medical microbiology lab, Department of Microbiology, KUST, Kohat, Pakistan, while the ELISA was performed at Mubarak Research and Diagnostic Lab, Peshawar, Pakistan.

### 2.2. Inclusion Criteria

The patients (different age groups and both genders) hospitalized in different wards who developed diarrhea after 2-3 days of hospitalization and who were on antibiotic therapy based on a predefined protocol were included in the study.

### 2.3. Exclusion Criteria

Patients with nondiarrheal stools without a history of any antibiotics and those where the cause of diarrhea (bacterial, viral, parasitic, and dietary) was diagnosed were excluded.

### 2.4. Patient and Public Involvement

No formal patient advisory committee was established, and there was no patient or public involvement in the design and planning of the study.

Data were collected on a questionnaire from all the subjects with preinformed consent regarding details of comorbidity if any (diabetes, hypertension, kidney or liver disease, and cancer), hospital admission, duration of hospital stay before diarrhea, colectomy, type of antibiotics used, duration, number and dose of antibiotics taken, use of proton pump inhibitors (PPI), and H2RA.

### 2.5. Culture of *C. difficile*

The stool samples were streaked onto cycloserine-cefoxitin fructose agar (CCFA) containing egg yolk and 5–10% defibrinated sheep blood and incubated anaerobically at 37°C for 48 hours. The AnaeroGen (Oxoid) gas-generator was used to generate anaerobic conditions. Identification *C. difficile* was performed by colony morphology, Gram staining, lecithinase/lipase activity, aerotolerance test, horse odor, and greenish-yellow fluorescence under UV of 365 mm wavelength light followed by biochemical tests such as indole and urease production, gelatin digestion, esculin hydrolysis and sugar fermentation tests, and final confirmation by the API 20A kit (bioMe´rieux). The pure isolates were preserved at −70°C in glycerol stock solution until further analysis.

### 2.6. Molecular Confirmation of *C. difficile*

For the confirmation of *C. difficile*, fresh colonies were used to extract genomic DNA using the phenol-chloroform extraction method and the extracted DNA was stored at −20 C for further molecular analysis. The tpi housekeeping gene using forward primer F: AAAGAAGCTACTAAGGGTACAAA and reverse primer R: CATAATATTGGGTCTATTCCTAC was amplified by polymerase chain reaction (PCR). The PCR protocol consisted of a predenaturation step at 94°C for 6 min, followed by 35 cycles of 30 sec at 94°C, 45 sec at 52°C, and 30 sec at 72°C. A final extension step was performed at 72°C for 10 min [15].

### 2.7. Drug Sensitivity Test and MIC Determination

Antibiotic susceptibility tests were determined using the Karby–Bauer disk diffusion assay against the commonly used antibiotics including ampicillin (AMP, 10 *μ*g), cefixime (CFM, 10 *μ*g), amoxycillin + clavulanic acid (AMC, 30 *μ*g), nalidixic acid (NA, 30 *μ*g), ciprofloxacin (CIP, 5 *μ*g), levofloxacin (LEV, 5 *μ*g), sulfamethoxazole + trimethoprim- (SXT, 1.25/23.75 *μ*g), vancomycin (VAN, 30 *μ*g), imipenem (IPM, 10 *μ*g), metronidazole (MTZ, 15 *μ*g), and chloramphenicol (CMP, 15 *μ*g) by the breakpoints as defined by the CLSI and for vancomycin as recommended by the European Committee on Antimicrobial Susceptibility Testing (EUCAST). The minimum inhibitory concentrations (MICs) of four antibiotics including AMP, MTZ, VAN, CIP, and CMP were determined by the agar dilution method according to the Clinical and Laboratory Standards Institute (CLSI) [22]. A previously characterized *C. difficile* strain obtained from the Department of Microbiology, KUST, was used as a control strain for the susceptibility tests.

### 2.8. Detection of Toxins in Stools by ELISA

The detection of toxins was conducted using ELISA. All positive stool samples were tested by an enzyme immunoassay toxin A/B kit (Creative Diagnostics, UK) for the presence of toxins A and B as per manufacturer protocol.

### 2.9. Molecular Detection of Toxins

PCR was carried out for the detection of the genes encoding toxin A and toxin B by specific primers; the toxin A gene primer tcdA F: AGATTCCTATATTTACATGACAATAT tcdA reverse R: GTATCAGGCATAAAGTAATATACTTT were used, while for the toxin B gene and tcdB F: GGAAAAGAGAATGGGTTTTATTAA and tcdB reverse R: ATCTTTAGTTATAACTTTGACATCTTT were used. The PCR reactions consisted of a predenaturation step at 95°C for 5 min, followed by 35 cycles of 1 min at 94°C, 40 sec at 52°C (for tcdA), 51°C (for tcdB), 53°C, and 45 sec at 72°C. A final extension step was performed at 72°C for 10 min. The PCR products were separated by electrophoresis in 2% agarose gel stained with ethidium bromide and visualized in GEL DOC XR + system (Biorad, USA).

### 2.10. Statistical Analysis

Data were analyzed by SPSS 2.0 software using chi-square and Fisher exact tests to check the association between the qualitative variables, as appropriate. *p* < 0.05 was considered significant.

## 3. Results

### 3.1. Frequency of *C. difficile* in Different Hospitals

**Figure 1:**
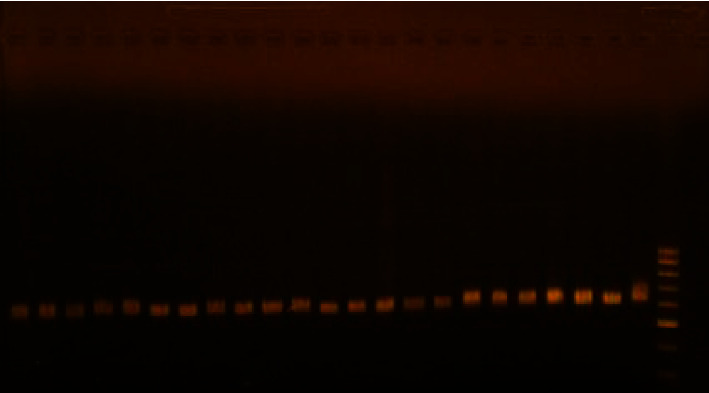
PCR amplicon of *TPi* gene (230 bp). Ladder (50 bp).

**Figure 2:**
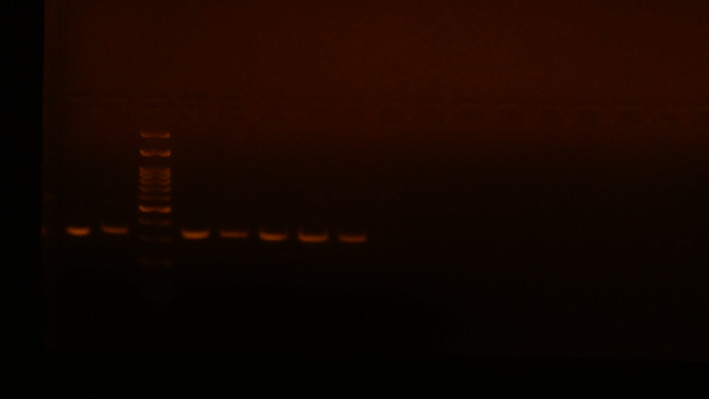
PCR amplicon (370 bp) *tcdA* gene (100 bp ladder).

**Figure 3:**
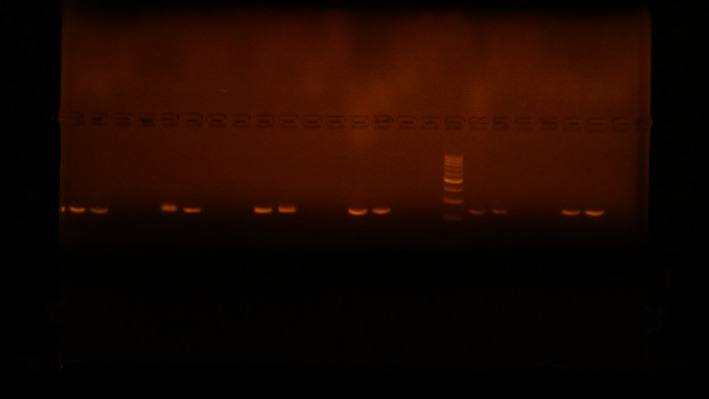
PCR amplicon (160 bp) *tcdB* gene (50 bp ladder).

In the present cross-sectional study, a total of 450 stool samples (150 from each hospital) were collected from patients hospitalized in three tertiary care hospitals of district Peshawar, KP, Pakistan. An overall frequency of 28.6% was recorded in these hospitals based on PCR results using Tpi gene (Table 1, Figure 1), PCR amplicon of *tcdA* gene (Figure 2), and *tcdB* gene (Figure 3). The highest frequency of *C. difficile* PCR-positive cases using Tpi gene was recorded from LRH (40.6% PCR-positive). Out of these 128 PCR-positive cases, 52.3% positive cases were recorded from the surgical ward, 28.5% cases in the medical ward, 12.3% cases in the ICU, and the remaining 6.7% cases were in the burn ward and oncology ward. No positive case was detected either at the urology or the gynecological ward. Out of these 128 positive cases, 58 (45.3%) were females and 70 (54.7%) were males. The age range of the patients was 20–85 years (mean of 42 years), while the mean age in the control group was 36 years. The highest CDI infection rate was noted for age group 20 (28.1%).

### 3.2. Antibiotic Susceptibility Profiles of *C. difficile* Isolates

The susceptibility of the isolates to different antibiotics was determined via the disc diffusion method as previously described [23] (Table 2). The highest susceptibility rate was found against vancomycin (*n* = 101, 93.7%%) followed by imipenem (*n* = 96, 88.8%) and levofloxacin (*n* = 72, 66.6%). The resistant rates among different isolates ranged significantly (*p* < 0.05) from 1.8 to 83.3% for other antibiotics, including metronidazole (*n* = 41, 38%, *p*=0.05), cefixime, and ciprofloxacin (51% each, *p*=0.06), cefepime (*n* = 60, 55.5% *p*=0.06), chloramphenicol (*n* = 57, 52.7%, *p*=0.07), amoxycillin (*n* = 81, 70%, *p*=0.06), and ampicillin (*n* = 90, 83.3%, *p*=0.18). Out of 108, 90.7% (*n* = 98) isolates were resistant to at least one antibiotic, and 90 (83.4%) were resistant to three or more antimicrobials.

### 3.3. MIC of Selected Antibiotics

The MIC ranges, MIC50 and MIC90, of vancomycin, metronidazole, ciprofloxacin, ampicillin, and imipenem against the isolates are shown in Table 3. The MIC90 of VAN was slightly higher than 2 mg·l^−1^, the susceptible category breakpoint of 2 mg·l^−1^. Therefore, only 2 (1.8%) vancomycin-resistant isolates were found. The MIC90 of ampicillin, ciprofloxacin, and metronidazole were considerably higher than the resistance breakpoints which indicate higher resistance against different classes of antibiotics. MIC50 and MIC90 of imipenem against the isolates were also above the breakpoint value which is reflected by the detection of imipenem-resistant isolates (*n* = 4, 3.7%, *p*=0.06).

### 3.4. Toxin Profile Detection by ELISA and PCR

Among 115 ELISA-positive strains, the highest frequency of toxin *A*+/*B*+ was found in 41% of the strains. The toxigenic profiles of *C. difficile* determined by ELISA are shown in Table 4.

### 3.5. Risk Factors Associated with *C. difficile* Infection

Overall, 72 (16.4%, *p*=0.003) diarrheal patients diagnosed with *C. difficile* infection had received cephalosporin and penicillin (33.3%, *p*=0.006), while 249 patients (34.8%) were on quinolones, and 111 (15.5%) patients were on different antibiotics during last 30 days. All of the patients having C. difficile-*associated* diarrhea had received antibiotics such as cephalosporin, ampicillin, amoxycillin, ciprofloxacin, metronidazole, vancomycin, and other antibiotics orally, while some patients had received antibiotics such as metronidazole and ciprofloxacin by intravenous route postsurgical interventions. There was a significant association of CDAD with age group (*p*=0.025), surgery for cholelithiasis (*p*=0.006), surgical intervention for colectomy (*p*=0.002), use of antibiotics (*p*=0.001), diabetes (*p*=0.007), hypertension (*p*=0.008), use of PPI (*p*=0.003), and H2 blockers' use (*p*=0.002) (Table 5).

## 4. Discussion

Emerging human pathogens in recent years caused devastating losses to the human population both economically and socially. Emerging pathogens need new strategies to overcome human losses and to avoid the spread of the pathogen during epidemic and pandemic eras. One of the important pathogens in recent decades is *C. difficile* which has emerged as a global threat to public health [24]. C diff infection (CDI) is becoming a major healthcare concern, as the severity of the disease is increasing steadily worldwide. This pathogen is considered the primary cause of intestinal infection associated with prolonged antimicrobial treatment, particularly in hospitalized patients [25, 26].

The present study was conducted to determine the incidence and risk factors of *C. difficile* associated with diarrhea patients in three major tertiary care hospitals in Peshawar, Pakistan. Our findings showed a high prevalence of CDI mainly within the age group of 20 (28.1%). These findings of CDI among the above age group are concurrent with a study conducted by Djuikoue et al. with a *similar infection rate* reported as having an almost similar prevalence rate (25.1%) [27].

Most emerging pathogenic bacteria are resistant to already available antibiotics, and health-related personnel need to find new solutions to avoid antibiotic resistance against specific pathogens. Therefore, we coupled the experiments by detecting the antibiotic profile of *C. difficile* infection followed by the detection of its toxins (A/B) by ELISA and PCR. The number of antibiotics that were used for the treatment of *C. difficile* infection is scarce, it is therefore important to obtain information about the resistant profile of CDI. In our study, we reported a significantly higher susceptibility rate against vancomycin (93.7%) followed by imipenem (88.8%) and levofloxacin (66.6%). The vancomycin group of antibiotics is the recommended antimicrobial used against the infection caused by *C. difficile* [28]. The previous study in Europe shows the high susceptibility of *C. difficile* to metronidazole and vancomycin against CDI, the common susceptibility of vancomycin in both studies states that the said antibiotic can be used as a drug of choice in treating CDI [29]. However, the resistance rate of metronidazole was 41% in the current study as opposed to the study previously explained. This unusual resistance level was caused probably by the indiscriminate use of metronidazole in less developed countries as it is one of the most frequently prescribed antibiotics against gastrointestinal infections. Two representatives of fluoroquinolones, i.e., levofloxacin and ciprofloxacin, were analyzed in the current study with resistance rates of 26% and 51%, respectively. In contrast to our study, a high resistance rate of ciprofloxacin was recorded in a study conducted by Tang et al. which can be attributed to overprescribing of the broad-spectrum antibiotics [30]. In addition to fluoroquinolones, high-rate resistance was observed in amoxycillin + clavulanic acid (81%); therefore, it should be limited in hospital settings to reduce the risk of CDI [27]. Similarly, the resistance rates of cephalosporins such as cefixime and cefepime are recorded as 51% and 60%, respectively, against CDI [31]. The MICs of five antibiotics including vancomycin, metronidazole, ampicillin, ciprofloxacin, and imipenem were determined. The majority of the strains were susceptible to vancomycin and imipenem with an MIC90 value of 2.5 mg/L and 3 mg/L. A study previously conducted in northern China by Wang and coworkers found all isolates' susceptibility to vancomycin and meropenem antibiotics. In the current study, only two isolates were found to be resistant to vancomycin and four strains were found resistant to imipenem. The highest susceptibility rate of these two antibiotics is nearly identical to the previous study conducted by Wang et al. [32].

The present study also includes the detection of important virulence factors that are involved in the pathogenicity of this emerging pathogen. Since CDI is a toxin-mediated disease and the expressions of its two toxins clostridia toxin A (TcdA) and B (TcdB) are the major causes of the symptoms, the groups of strains (*A* + *B*+, *A* + *B*− and *A* − *B*+) as defined in Table 4 of *C. difficile* were defined based on the possession of the toxigenic genes such as *tcdA* and *tcdB*, but their detection rate in various clinical incidences is different with the most common toxigenic type as *A* − *B*− [33]. An EIA immunoassay and PCR were performed for the detection of toxin enzymes and genes in addition to the stool culture method. Our result shows high sensitivity and specificity for PCR (28.6%) as compared to the other two methods used, EIA (25.5%) and stool culture (24%). The current finding was parallel to the finding of Elgendy and his coworkers who show that the use of direct PCR is considered a specific and sensitive tool as compared to other methods employed [34, 35]. Therefore, sensitive molecular detection is an important step in finding the exact pathogen and its toxin in diseased patients.

We realize that there are a few limitations to this study. First, the study includes the tertiary care hospital of one district, particularly Peshawar. Second, we have not categorized patients with low- and high-risk groups of diarrheal patients. Lastly, the heterogeneity of patients was not determined among patients admitted to various medical wards. Therefore, the study is required to determine the limitations observed in the current study.

Despite the limitations, the overall study concluded that the infections caused by emerging human pathogens like *C. difficile* are usually alarming, and novel strategies may opt for a better antibiotic regime to avoid growing antibiotic resistance and diagnostic procedures that will certainly reduce the burden of CDI in the community.

## Figures and Tables

**Table 1:** Incidence of *C. difficile* infection in different hospitals of Peshawar by culture, EIA, and PCR.

Hospital	Total samples *n* (%)	Culture positive *n* (%)	EIA toxin *A*/*B* positive *n* (%)	PCR positive *n* (%)
HMC	150	30 (27.5)	36 (31.1)	41 (32)
LMH	15	47 (44.1)	49 (42.6)	52 (40.6)
KTH	150	31 (28.4)	33 (28.3)	35 (27.2)
Total	450	108 (24)	115 (25.5)	128 (28.6)

**Table 2:** Phenotypic antimicrobial susceptibility patterns among *C. difficile* strains (*n* = 108) using the disc diffusion assay.

Antibiotic	Code	Concentration (*μ*g)	S *n* (%)	I *n* (%)	R *n* (%)
Ampicillin	AMP	10	12 (11.1)	6 (5.6)	90 (83.3)
Cefixime	CFM	10	30 (27.7)	27 (9.2)	51 (47.2)
Cefepime	CEF	30	38 (35.1)	10 (10.4)	60 (55.5)
Amoxycillin + clav. acid	AMC	30	10 (13.8)	22 (19.4)	81 (70)
Nalidixic acid	NA	30	27 (25)	15 (13.8)	66 (61.1)
Ciprofloxacin	CIP	5	35 (13.8)	22 (20.3)	51 (47.2)
Levofloxacin	LEV	5	72 (66.6)	10 (9.4)	26 (24)
SXT	SXT	25	32 (30)	16 (13)	60 (55.5)
Chloramphenicol	CMP	30	45 (41.6)	6 (5.2)	57 (52.7)
Imipenem	IPM	10	96 (88.8)	8 (7.4)	4 (3.7)
Vancomycin	VAN	10	101 (93.5)	—	7 (6.4)
Metronidazole	MTZ	15	53 (21.2)	14 (6.4)	41 (38)

S = sensitive, I = intermediate, R = resistant.

**Table 3:** MIC of different antibiotics against *C. difficile* isolates.

Antibiotics	MIC range (mg/lit)	MIC50 (mg/L)	MIC90 (mg/L)	Clinical breakpoints			S *n* (%)	I *n* (%)	R *n* (%)
				S	I	R			
Vancomycin	0.06–2	1	2	≤2	—	>2	101 (93.5)	—	7 (6.4)
Metronidazole	0.015–0.5	0.5	0.5	≤2	—	>2	53 (21.2)	14 (6.4)	41 (38)
Ampicillin	0.03–2	0.5	2	≤4	8	≥16	12 (11.1)	6 (5.6)	90 (83.3)
Ciprofloxacin	0.12–30	2.0	30	≤2	4	≥8	35 (13.8)	22 (20.3)	51 (47.2)
Imipenem	0.25–4	2.5	4	≤4	8	≥16	96 (88.8)	8 (7.4)	4 (3.7)

**Table 4:** Frequency of toxigenic strains of *C. difficile* based on *A* and *B* toxins ELISA.

Total samples *n* (%) positive by ELISA	No. of positive samples *n* (%)	Toxin status	Toxin *A* ELISA	Toxin *A*/*B* ELISA
115/450 (25.5)	41 (35.6)	*A*+/*B*+	+	+
	23 (20)	*A*−/*B*+	−	+
	51 (44.4)	*A*−/*B*−	−	−

**Table 5:** Risk factors associated with *C. difficile* associated diarrhea in hospitalized patients.

Risk factors	(*n* = 128)	*p* value
Age group (60–80 years)	48 (15.5%)	<0.002
Age groups below 60 years	80	
Gender: male	83	
Female	45	
Hospitalized recently	102	
Not hospitalized recently	26	<0.0001
Antibiotics used in last 2 weeks	113	
Antibiotics not used	15	
Proton pump inhibitors (PPI) used	87	<0.0003
PPI not used	41	
Chemotherapy used	42	
Chemotherapy not used	86	<0.001
H2 blockers used	68	
H2 blockers not used	60	
Diabetes positive	71	0.007
Diabetes negative	57	
Hypertension present	82	0.006
Hypertension absent	46	
Kidney disease present	47	
Kidney disease not present	81	
Liver disorder present	51	
Liver disorder absent	77	0.008
Any kind of malignancy present	21	
Malignancy absent	105	
Cholelithiasis surgery	45	0.007
Surgery not done	83	
Colon surgery done	24	
Colon surgery not done	104	

## Data Availability

All the data generated and analyzed are included in the article.

## Abbreviations

PCR: Polymerase chain reaction
ELISA: Enzyme-linked immunosorbent assay
*C. difficile*: *Clostridium difficile*
CDI: *C. difficile* infection
MDR: Multidrug-resistant
MIC: Minimum inhibitory concentrations.
